# Micropropagation of an Exotic Ornamental Plant, *Calathea crotalifera*, for Production of High Quality Plantlets

**DOI:** 10.1155/2014/457092

**Published:** 2014-07-20

**Authors:** Shahril Efzueni Rozali, Kamaludin A. Rashid, Rosna Mat Taha

**Affiliations:** ^1^Institute of Biological Sciences, Faculty of Science, University of Malaya, 50603 Kuala Lumpur, Malaysia; ^2^Center for Foundation Studies in Science, University of Malaya, 50603 Kuala Lumpur, Malaysia

## Abstract

A successful protocol was established for micropropagation in two selected varieties of exotic ornamental plants, *Calathea crotalifera*. The effects of different sterilization techniques, explant type, and the combination and concentration of plant growth regulators on shoots induction were studied. The axillary shoot buds explants sprouted from rhizomes in soil free conditions showed high induction rate of shoots with lowest contamination percentage when treated with combination of 30% (v/v) NaOCl, 70% (v/v) ethanol, and 0.3% (w/v) HgCl_2_. In the present study, the highest number of multiple shoots was obtained in MS basal medium supplemented with 3.5 mg/L 6-Benzylaminopurine (BAP), 1.0 mg/L 1-Naphthaleneacetic acid (NAA), 3% sucrose, and 6 g/L plant agar for both varieties and was used as multiplication medium. Microshoots were highly induced when the young shoot bud explants were incised longitudinally prior subculture. Chlorophyll analysis was studied to test the effects of activated charcoal and L-glutamine on reduction of necrosis problem. The maximum roots induction was recorded on MS medium supplemented with 1.0 mg/L 1-Naphthaleneacetic acid (NAA) compared to indolebutyric acid (IBA). The complete regenerated plantlets were successfully acclimatized in the soilless medium under greenhouse condition. This is the first report of rapid mass propagation for *C. crotalifera*.

## 1. Introduction

*Calathea crotalifera* is one of the important ornamental plants belonging to Marantaceae family. This rhizomatic plant species can produce a very attractive inflorescence and have been used in landscaping especially for screening and indoor plants. The other* Calathea sp.* has also been used widely in horticulture industry due to its attractive foliar colors and variegation patterns [1]. The exotic appearance of* C. crotalifera* inflorescence which is known as the rattle shaker flower for the flower's resemblance to a rattlesnake's rattle was making it more popular in cut flower industry. The inflorescence has attractive bracts (modified leafs) that can be found in four different colors which are red, white, green, and yellow with a few conspicuous flowers that will peek out from the bract as they mature. The vase life of the inflorescence can vary greatly like the other exotic tropical ornamentals such as* Heliconia* sp.,* Strelitzia* sp., and* Alpinia* sp. [2].* C. crotalifera* was traditionally propagated through seeds and by vegetative techniques using the cutting of rhizomes. However, this propagation technique shows very slow growth rate which is lengthy for large-scale multiplication. An efficient multiplication method is required to supply sufficient disease-free plant for large scale cultivation using* in vitro* propagation. The production of microcuttings with high quality plantlets that are suitable for transplanting into the natural environment was especially in demand. There has been scant information on* in vitro* propagation of Marantaceae [3, 4]. A few studies on* in vitro* propagation of Marantaceae have already been reported, for example,* Calathea ornata* Koern [5],* Calathea orbifolia* (Linden) Kennedy [1], and* Maranta leuconeura* cv. Kerchoviana [6]. However, to date, there were no reports on the efficient micropropagation of* C. crotalifera* which had been performed in the present study. The aim of this study was to develop and establish an aseptic technique in micropropagation method of red and yellow* C. crotalifera* and to induce the formation of multiple shoots and also to produce a high quality regenerated plantlets which can be successfully transferred to the soil.

## 2. Materials and Method

### 2.1. Establishment of Aseptic Explants

**Figure 1:**
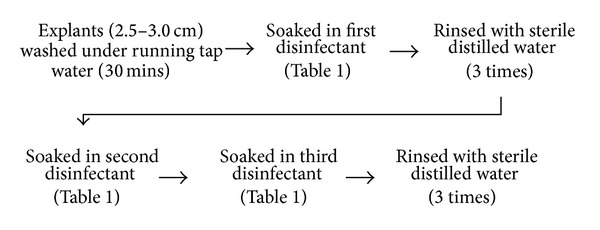
General sterilization method for shoot bud explants of* Calathea crotalifera*.

Mature plants of red bract* C. Crotalifera* (CCR) and yellow bract* C. Crotalifera* (CCY) were purchased from local farm, Agrobiosolution Company located in Kuantan, Pahang, Malaysia. The plants were maintained at planting area at Centre for Foundation Studies in Science, University of Malaya, under natural condition. Two different sources of young shoot buds were used as explants in this study. The first source of explants is apical young shoot buds obtained directly from the mature plants planted in the soil. The second source of explants was obtained from the sprouting axillary young shoot buds that appeared from the cleaned rhizomes after two weeks under soil free conditions. Suitable explants were collected and washed with detergent under running tap water to remove any soil particles attached to the shoot buds. The external leaves were removed and the shoot buds were trimmed down until the size ranged from 2.5 to 3.0 cm. The explants were then rinsed under running tap water for 30 minutes. Under aseptic conditions, the explants were surface sterilized with three different treatments as shown in Table 1 by following the general sterilization method summarized in Figure 1. Each disinfectant (30% sodium hypochlorite, NaOCl, 70% ethanol, and mercury chloride, HgCl_2_) was added together with two to three drops of Tween-20 to reduce surface tension. The surface sterilized explants had their external leaves removed and were finally dried on filter papers in the laminar flow air chamber prior to inoculation on media. The contamination, necrotic, and survival percentages of cultures were determined as in Sundram [7].

### 2.2. Determination of Culture Media for Multiple Shoots Induction

The surface sterilized shoot buds explants were trimmed down until the size ranged from 1.0 to 1.5 cm. They were then inoculated on a MS [8] basal medium containing 30.0 g L^−1^ sucrose with different concentrations of plant agar and combinations of plant growth regulators (Tables 2, 3, and 4). Axillary shoot bud explants were used in the study on effects of different agar concentrations on shoot production. The explants were inoculated on MS medium supplemented with 3.0 mg/L BAP and 1.0 mg/L NAA. Media were adjusted to pH 5.8 prior to autoclave at 121°C for 15 minutes. The cultures were maintained in the growth room with 16/8 h photoperiod at 25 ± 2°C. In the present study, 10 replicates were used and subculturing was carried out at an interval of eight to twelve weeks. All cultures were examined periodically and the morphological changes were recorded after six weeks of cultures. The effects of different media treatments were determined and quantified based on the percentage of explants showing response for multiple shoots induction. The new shoots produced were used and subcultured for the next subsequent experiments.

### 2.3. Effect of Apex Damage to Induce Microshoots Formation

The effects of apex damage on induction of microshoots were determined based on the method suggested by Nathan et al. [9] and Podwyszyńska [5]. The three months* in vitro* shoot of CCR and CCY that have been induced in the multiplication media was used. Two techniques of apical dominance elimination were applied in the present study. The first shoot was incised horizontally about 0.5–1.0 cm over a shoot base whereas the second shoot was incised by halving the buds longitudinally. These explants were then subcultured on the optimum multiplication media. The cultures were incubated under the same culture condition and the data were recorded and analysed in the same manner.

### 2.4. Effects of Glutamine and Activated Charcoal on Production of High Quality Plantlets

According to the obtained results for multiplication shoots induction in the first study, most of the plantlets showed the presence of necrotic and hyperhydricity leaves. Addition of different concentrations of glutamine and activated charcoal in the growing media (Table 6) was used to improve the shoot quality with low percentage of necrotic leaves. The three months* in vitro* shoot of CCY was used and the shoot was incised prior to culture in the different media treatments. The data was collected after two months cultured and analyzed based on the percentage of necrotic leaves and concentration of chlorophyll pigments in the leave. Method for pigment identification proposed by Slatnar et al. [10] with a few modifications was used in the present study. For chlorophyll pigment analysis, leaf disc with 1 cm^2^ area was mashed in 1 mL DMSO with addition of calcium carbonate powder in the 1.5 mL microtube. The extracts were incubated in the water bath for 2 hours at 60°C prior to centrifugation for 5 minutes at 5000 g. The collected supernatant was then finally analyzed using UV-spectrophotometer at 648 nm (Ch b), 666 nm (Ch a), and 480 nm (carotenoid). The concentration of each pigment was calculated using Wellburn Equation [11].

### 2.5. Effects of Auxins on Roots Induction

*In vitro* shoots of yellow CCY from six months old stock cultures were used for* in vitro* roots induction. Shoots were separated from their clumps and cultured individually in full strength MS medium containing 30.0 g L^−1^ sucrose and augmented with different concentrations of IBA or NAA at 0.0, 0.1, 0.5, 1.0, and 1.5 mg L^−1^ which added separately (Table 8). The medium was adjusted to pH 5.8 and solidified with 6.0 g L^−1^ plant agar. The effects of auxin were determined by measuring the root length, root diameter, and root number of each treatment after 60 days of incubation.

### 2.6. Acclimatization and Morphological Development of Leaf Surface

Complete plantlets with high quality shoots and roots (Figures 4(g) and 4(h)) were removed from the rooting medium and the roots were carefully cleaned under running tap water to remove the residual agar. Each plantlet was then transplanted into plastic pots containing mixture of black soil, river sand, coconut husk, and vermiculite (4 : 2 : 2 : 1). The plantlets were then allowed to grow for two months under greenhouse conditions at 25 ± 2°C with direct sunlight for 12 h daily. Each pot was watered with distilled water for everyday and was covered with transparent plastic which was gradually removed. The morphology of leaf stomata from two months acclimatized plantlets were examined under scanning electron microscope (SEM). The leaf disc with 0.25 cm^2^ area from the* in vitro* and* in vivo* grown plantlets was used to compare the morphological development of the leaf surface and leaf stomata from two different conditions. The samples were fixed in 2% aqueous osmium tetroxide, OsO_4_ for overnight at 4°C. The treated samples were rinsed with distilled water for two times at 15 minutes each prior to dehydration through ethanol series (10, 20, 30, 40, 50, 60, 70, 80, 95, and 100%). The samples were then infiltrated through ethanol, acetone mixture at 15 minutes each, and ended with pure acetone for 1 hour before dried using a Critical Point CO_2_ Dryer. The samples were finally viewed under SEM after being gold coated. The presence of wax and morphological development of stomata aperture on the abaxial and adaxial leaf surfaces were observed and recorded.

## 3. Statistical Analysis

The data collected were analyzed by one way ANOVA and the mean values ± SE were subjected to statistical analysis using Duncan's multiple range test (DMRT) at 5% significance level.

## 4. Results and Discussion

### 4.1. Establishment of Aseptic Explants

**Figure 2:**
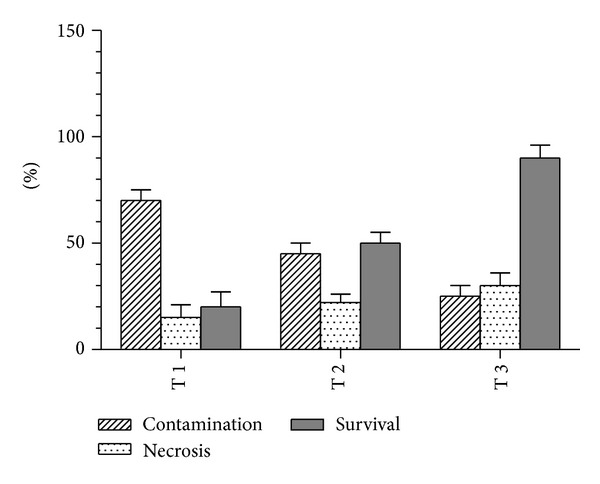
Percentage of surviving explants under different treatments in surface sterilization.

Two different types of young shoot bud explants were used in this study, which are apical shoot bud from soil medium and axillary shoot bud from soilless medium. Both of these explants were surface sterilized using the same protocol based on Figure 1 and Table 1, respectively. In the present study, axillary shoot buds showed the lowest percentage of contamination (25%) when compared to apical shoot buds (60%). Thus, comparison of the effectiveness in three different surface sterilization treatments (T1, T2, and T3) on axillary shoot bud explants was used in this study. The best treatment of surface sterilization was achieved when 30% NaOCl, 70% ethanol, and 0.3% HgCl_2_ were utilized in T3 with the lowest percentage of contamination (25 ± 5%) compared to T1 and T2. Furthermore, percentage of surviving explants was higher in T3 (90 ± 6%) followed by T2 and T1 even though the percentage of necrotic leaf in T3 is higher compared to other treatments. Most of the contamination is caused by bacteria and no fungi infections were found. Establishment of contamination free cultures was a major task since the explants were taken from the underground rhizomes [12]. The using of axillary bud that initially sprouted in soil free condition can actually reduce the potential of the explants to be infected by the soil bacteria. This method has been used widely in micropropagation of other rhizomatic species like* Heliconia psittacorum* [9],* Boesenbergia rotunda* L. [13], and* Curcuma mangga* [7]. Disinfecting with 70% ethanol prior to soaking in HgCl_2_ enhances the contact between HgCl_2_ and the surface of explants efficiently. Smith [14] reported that mercury ions in HgCl_2_ solution can break the structure of the cell membrane and the cytoplasm constituents of pathogenic microorganisms by interfering the enzymes and also the protein molecules. The concentration of HgCl_2_ was not increased more than 0.3% as in T3 because the high concentration of HgCl_2_ is phytotoxic to plant cells [7]. Based on the results, T3 was the most effective method with the highest survival rate (Figure 2) after four weeks of cultured. This treatment was therefore applied for subsequent experiments.

### 4.2. Effects of Medium Solidification on Shoot Induction

**Figure 3:**
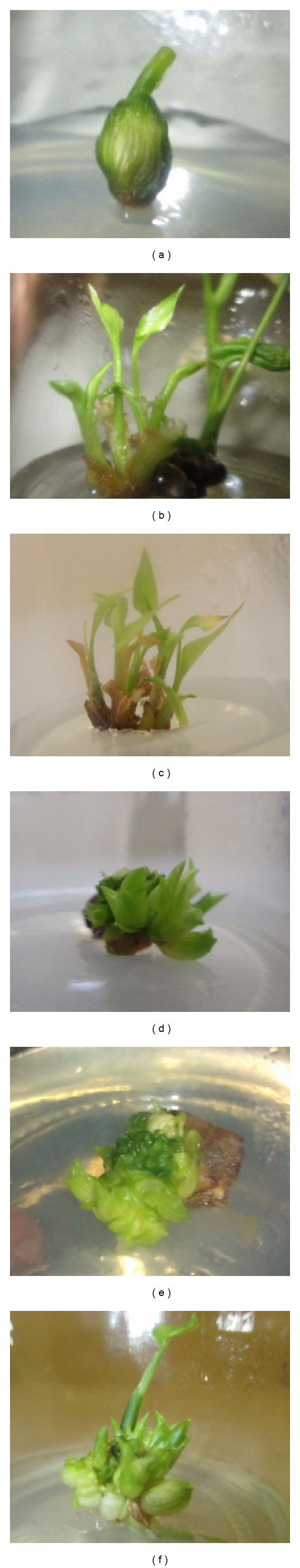
Effects of medium solidification and apex damage. (a) Swollenness of explant in media with 3 g/L plant agar. (b) Leaves show vitrification problem in media with 3 g/L plant agar, (c) regenerated shoots with necrotic leaves in media with 8 g/L plant agar. (d), (e), and (f) Induction of microshoots through elimination of apical dominance.

The using of different concentrations of agar (0.3%, 0.6%, and 0.8%) had showed significant effects on shoot production as shown in Table 2 after eight weeks of culture. The number and height of shoot produced in media augmented with 3.0 g/L and 8.0 g/L g of plant agar was lower for both species of* C. crotalifera*. Development of the shoots produced in these respective media was not in good condition which showed vitrification and browning problem as shown in Figure 3. The best concentration of plant agar for shoot induction in the present study was 6.0 g/L and this concentration was used in the next multiplication media. The similar application has been reported in induction of shoot of* Maranta leuconeura* cv. Kerchoviana on medium augmented with 6 g/L plant agar [6]. Vitrified leaves observed in the present study were caused by high relative humidity [15] and can be reduced using high concentration of gelling agents [16]. Kataeva et al. [17] reported that the vitrification problem has been correlated to water availability, microelements, and hormonal imbalance in the tissue culture medium. Vitrification or well known as hyperhydricity is a morphological, anatomical, and physiological malformation that makes the plant tissue water-swollen [18] and the* in vitro* plantlets will have poor epicuticular wax production [19].

### 4.3. Effects of Cytokinin and Auxin on Shoot Organogenesis from Two Different Types of Explants

In general,* in vitro* culture of apical and axillary shoot bud explants on MS medium supplemented with auxin and cytokinin started to swell up after two weeks of culture. Tables 3 and 4 show the effect of different concentrations and combinations of NAA, BAP, and Kinetin on shoots organogenesis from two different types of explants. Shoot organogenesis from axillary shoot bud explants was faster (within four weeks) compared to apical shoot bud explants (after eight weeks). The different of this growth rate caused the shoots produced from axillary shoot buds explants were higher compared to apical shoot bud explants in both varieties, CCY and CCR after two months culture. Similar result was obtained in rapid multiplication of* Boesenbergia rotunda* L. by using sprouted axillary bud as initial explants [13]. Based on both results, the production of shoot was increased as the concentration of BAP and Kinetin was increased. However, the number of shoot productions was reduced when the concentration of BAP was higher than 3.5 mg/L. The highest number of shoot productions in CCY (5.60 ± 0.51) and CCR (4.40 ± 0.24) was obtained from axillary shoot bud explants in MS medium supplemented with 3.5 mg/L BAP and 1.0 mg/L NAA with highest height of shoot, 5.44 ± 0.06 cm and 4.85 ± 0.12 cm, respectively. Furthermore, the percentage of leaf production per explants in CCR and CCY from axillary shoot bud explants was also higher compared to apical shoot bud explants. These results demonstrated that combination of cytokinin and auxin can give significant effects on shoot induction from apical and axillary shoot bud explants in both* Calathea* sp. Podwyszyńska [5] reported the establishment of optimum shoot multiplication of* C. ornate* Koern on MS medium supplemented with 2.5 mg/L BAP and 2.5 mg/L Kinetin. Different results were obtained in other Marantaceae plants as in multiple shoot induction of* Maranta leuconeura* cv. Kerchoviana was achieved by culturing the shoot tip explants on MS medium supplemented with 5 mg/L BAP [6] or when the lateral buds were cultured on Linsmaier and Skoog medium supplemented with 0.2 mg/L BAP [3]. Present study showed that further increase of BAP concentration will reduce the number of shoots induction and can cause necrosis (result not presented), indicating an adverse effect of plant growth regulators beyond the optimal concentration [20]. Higher concentration of cytokinin beyond the optimum levels was also reported to cause necrosis and reduction in shoot formation during* in vitro* multiplication of* Musa* sp. [21, 22]. Shoot organogenesis and multiple shoots induction by using various concentrations of BAP and NAA had been reported in several micropropagations of ornamental rhizomatic plants like* Alpinia purpurata* [23],* Costus pictus* D. Don [24],* Heliconia psittacorum* [9], and* Musa beccarii* [25]. The overall results in the present study showed that the using of axillary shoot bud explants in production and development of new shoots was more effective compared to the using of apical shoot bud explants.

### 4.4. Effects of Apex Damage to Induce Microshoots Formation

Stimulation of microshoots was obtained through elimination of apical dominance by shoot incision after 12 weeks culture (Table 5). Two types of incision were applied in this method to determine the effects on the microshoots production. Based on the results, microshoots were highly produced from the shoot bud that was longitudinally incised prior to culture. This result revealed that this method can be applied in mass propagation in order to increase the multiplication rate. Similar technique was used in stimulation of shoot branching in* Strelitzia* sp. [26] and* Calathea ornata* [5]. The excision of an apex is important for mass propagation of valuable ornamental plants with a naturally low rate of multiplication like* Strelitzia* sp. and* Calathea* sp. Cronauer and Krikorian [27] also reported the similar findings in induction of multiple shoots of two dessert banana clones and two plantain clones through excision of shoot tips. This phenomenon had been discussed precisely by Shimizu-Sato et al. [28] as it was found that cytokinin will be induced by decapitation of the shoot apex and stimulate axillary bud outgrowth.

### 4.5. Production of High Quality Plantlets through Addition of Glutamine and Activated Charcoal

**Figure 4:**
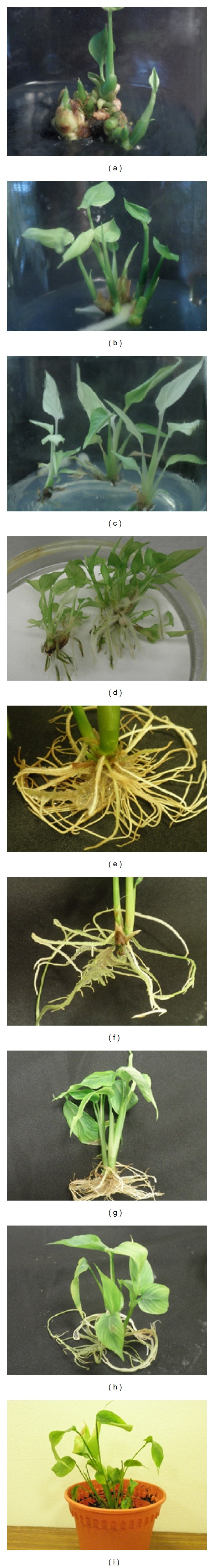
Development of high quality plantlet from shoot bud explants of* Calathea crotalifera* on multiplication media (MS medium with 3.5 mg/L BAP and 1.0 mg/L NAA) and rooting media (MS medium with 1.0 mg/L NAA). (a) Induction of microshoots from incised shoot bud explants. (b) High quality plantlets with dark green leaves in multiplication medium supplemented with 1 g/L activated charcoal. (c) Plantlets with light green leaves without necrosis problem on multiplication medium supplemented with 1 g/L L-glutamine. (d) Plantlets with high multiple shoots induction. (e) Induction of healthy root in rooting media supplemented with 1.0 mg/L NAA. (f) Roots induction on rooting media supplemented with 1.0 mg/L IBA. (g) and (h) Healthy plantlets ready to be acclimatized. (i) Development of successfully acclimatized plantlets.

This study was carried out to reduce necrosis problems presence in the previous culture through addition of glutamine, activated charcoal, or their combinations.  Figure 4 and Table 7 show the stages of shoot development and the results obtained on leaf pigment concentration after three months of culture, respectively. Three types of leaf pigments were evaluated in this study, which are chlorophyll a, chlorophyll b, and carotenoid. Results show that the chlorophyll concentration in the leaf samples was varied depending on concentrations of glutamine and activated charcoal enriched in the medium. Addition of activated charcoal in MS medium was the most effective for production of shoots with dark green leaf and containing high concentration of chlorophyll pigments. However, the percentage of necrotic leaves was proportionally increased with the increasing of activated charcoal concentration. Multiplication medium fortified with glutamine produced shoots with light green leaf (Figure 4(c)) and containing low concentration of chlorophyll pigments and shows the lowest percentage of necrotic leaf. This result revealed that exogenous amino acid from glutamine serves as nitrogen source for the synthesis of protein [29] which is important to reduce necrosis effect on the leaf. In addition, Zouine and Hadrami [30] reported that exogenous supply of glutamine can possibly increase soluble storage protein in embryogenic cells of date palm suspension culture. Based on the overall result, the high quality* in vitro* plantlets with dark green leaf and low percentage of necrotic leaf were observed on multiplication medium fortified with 1.0 mg/L activated charcoal (3510 1C), (Table 7). Activated charcoal plays an important role in* in vitro* morphogenesis due to its irreversible adsorption of inhibitory compounds in the culture medium and substantially decreasing the toxic metabolites, phenolic exudation, and brown exudate accumulation, which also can promote growth and adsorption of vitamins, metal ions, and plant growth regulators, including gaseous ethylene [31]. Activated charcoal had been used to reduce explant browning problem in shoot tip culture for cryopreservation protocols in* Rubus idaeus* [32] at concentration of 0.25 g/L fortified in MS medium. The explant browning also could be overcome by growing embryos of* Dipterocarpus alatus* and* D. intricatus* initially on a filter paper bridge in liquid medium with activated charcoal to absorb the oxidized phenolic compounds [33]. Figures 3(d), 3(e), and 3(f) show the induction of multiple shoots and rapid shoot elongation from the incised shoot buds in 3510 1C medium. The similar observations on induction and elongation of shoots have been reported in micropropagation studies such as cashew [34], eucalyptus [35], lilly [36], cotton [37], and yam [38]. Roots were also produced from the two to three months old plantlet of CCY and CCR cultured in the 3510 1C medium without transferring in the rooting medium (Figure 4(b)). Several studies have demonstrated that addition of activated charcoal alone or combined with auxin can promote roots induction from the mature* in vitro* plantlets [39–43]. Apart from that, according to Eymar et al. [44], activated charcoal also can give significant effect on* in vitro* nitrogen uptake in* Lagerstroemia indica* which demonstrated that the explants grown in medium with activated charcoal were capable of taking up both NO^3−^ and NH^4+^. Similar results were showed in the present study where the multiplication medium fortified with 1 g/L glutamine and 1 g/L activated charcoal (3510 G1C) produced high quality of plantlets as in 3510 1C medium (Table 7).

### 4.6. Root Induction

Based on results in Table 8, interaction of type and hormone concentration had significant relation with length, diameter, and number of roots. In general, roots were induced after three weeks of culture. Root number in both rooting media with NAA and IBA, respectively, was increased as the concentration of the auxin increased and started to decline when the concentration was higher than 1.0 mg/L. Media with low concentration of auxin produced longer roots with small size in diameter. This result demonstrates that the plantlets could be rooted in both types of auxins but the highest number of roots was produced in MS medium supplemented with 1.0 mg/L NAA. Plantlet that cultured in media fortified with several concentrations of NAA produced high quality of roots in terms of number of roots, root length, and root diameter (Figure 4(e)). Similar results were reported by Hamad et al. [45] where media enriched with 1.0 mg/L NAA were the best media for induction of adventitious roots in pineapple and produced tallest plantlets with high number of roots per shoot. Raihana et al. [46] also demonstrated that MS medium supplemented with 1.0 mg/L NAA gave the highest root number in micropropagation of* Curcuma mangga* from rhizome bud where the increasing of NAA could suppress the production of root. However, Loc [47] and Yusuf et al. [13] reported that MS medium supplemented with 2.0 mg/L NAA could enhance roots induction in* Curcuma zedoaria* and* Boesenbergia rotunda*, respectively. Several studies demonstrated that most of the micropropagated rhizomatic plants can produce roots in MS medium devoid of auxin with or without addition of activated charcoal like* Musa* sp. [42, 48, 49],* Curcuma* sp. [50],* Zingiber* sp. [51], and* Heliconia* sp. [9]. The study suggested that these results might be due to the fact that some of rhizomatic plants can produce sufficient amount of auxin endogenously to initiate root induction.

### 4.7. Micromorphological Development of Leaf in Acclimatized Plantlets

**Figure 5:**
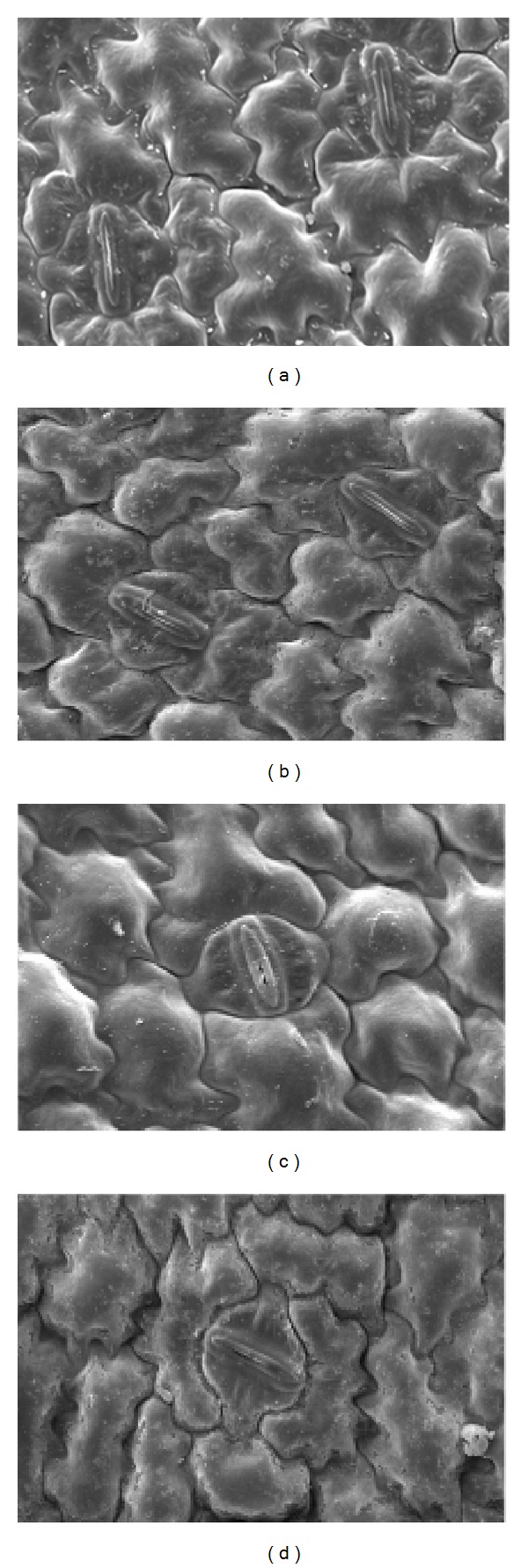
Scanning electron micrograph showing abaxial (a) and adaxial (c) surfaces of* in vitro* leaves of plantlet of* Calathea crotalifera*, abaxial (b) and adaxial (d) surfaces of leaves from* in vivo* (acclimatized) plant.

The most crucial phase in micropropagation study is acclimatization of complete* in vitro* plantlets to the natural environment. Healthy plantlets taken out from the* in vitro* condition usually produced leaves with low epicuticular wax and with ineffective control of stomatal function [52]. These phenomena promote water loss through transpiration when the plantlets are transferred to the soil. In the present study, high quality of* in vitro* plantlets of* C. crotalifera* was successfully acclimatized with 75% of plantlet survival after four weeks of transfer (Figure 4(i)). In general, there were no morphological differences in the vegetative characters between* in vivo* and* in vitro* plant of* C. crotalifera* except for the sizes and texture of leaves. The texture of* in vitro* leaves surface was membranous while* in vivo* leaves were coriaceous due to the presence of thick cuticle layer with the presence of wax on* in vivo* leaves and absence of it on* in vitro* leaves as shown in Figure 5 under scanning electron microscope. Based on this micromorphological study, the acclimatized plantlets were well developed to adapt with the natural environment since they were exposed to the sun. The presence and thickness of the cuticle is dictated by environmental factor including sunlight and can be an indicator for climate and habitat [53]. The difference of cuticle thickness between* in vitro* and* in vivo* leaves has been reported in acclimatization of micropropagated of* Murraya paniculata* plantlets [54]. The leaf lamina of* in vitro* and* in vivo* of* C. crotalifera* also showed little differences on anatomical characters since both leaves were hypostomatic. The mean number of stomata was higher on the abaxial surface compared to adaxial surface of the leaves (Table 9). This anatomical characteristic was also found on* C. orbifolia* (Linden) leaves as reported by Yang and Yeh [1].

## 5. Conclusion

Sterile explants from shoot buds of* Calathea crotalifera* (yellow and red bract) could be established using combination of 30% NaOCl (15 minutes), 70% ethanol (1 minute), and 0.3% HgCl_2_ (10 minutes). This study indicates that rapid shoot induction has been obtained from axillary shoot bud explants cultured in the MS medium supplemented with 3.5 mg/L BAP and 1.0 mg/L NAA, with 6 g/L of plant agar. Microshoots were highly produced from the shoot bud that was longitudinally incised prior to culture in the multiplication media due to the elimination of apical dominance. Production of high quality and healthy vigorous plantlets with low necrosis problem and vitrified shoots, and also with a higher chlorophyll content, have been established in the multiplication medium enriched with 1 g/L activated charcoal and glutamine. The overall results demonstrated that this protocol is cost effective in terms of reduction of explants contamination percentage and production of multiple shoots in a short period. Microshoots and* in vitro* plantlets can be an important source of free disease planting material, ideally suited for germplasm exchange, transportation, and conservation of this exotic ornamental plant species.

## Figures and Tables

**Table 1:** Three different treatments in surface sterilization for shoot bud explants of *C. crotalifera*.

Disinfectant	Treatment 1 (T1)	Treatment 2 (T2)	Treatment 3 (T3)
First disinfectant (v/v)	30% NaOCl (15 minutes)	30% NaOCl (15 minutes)	30% NaOCl (15 minutes)
Second disinfectant (v/v)	30% NaOCl (10 minutes)	70% ethanol (1 minute)	70% ethanol (1 minute)
Third disinfectant (w/v)	70% ethanol (1 minute)	0.1% HgCl_2_ (10 minutes)	0.3% HgCl_2_ (10 minutes)

**Table 2:** Effects of different agar concentrations on shoot production of *C. crotalifera *(CCR and CCY).

Concentration of plant agar (g/L)	Number of shoot**s**		Height of shoot (cm)		Overall observation
	CCY	CCR	CCY	CCR	
3.00	1.00 ± 0.0^a^	1.00 ± 0.0^a^	1.96 ± 0.19^a^	1.80 ± 0.12^a^	Swollen with vitrified shoot
6.00	1.80 ± 0.37^b^	1.90 ± 0.24^b^	3.48 ± 0.14^b^	3.44 ± 0.10^b^	Swollen with green shoot
8.00	1.00 ± 0.0^a^	1.00 ± 0.0^a^	2.30 ± 0.13^a^	2.00 ± 0.16^a^	Swollen with browning shoot

Mean ± SE, *n *= 10. Different letters indicate significant differences between media at *P *= 0.05.

**Table 3:** Effects of different concentrations and combinations of cytokinin and auxin on shoot production from two different sources of explants in yellow *C. Crotalifera *(CCY).

BAP (mg/L)	Kinetin (mg/L)	NAA (mg/L)	Number of shoot**s**		Height of shoot (cm)		Percentage of leaf (%)	
			Apical	Axillary	Apical	Axillary	Apical	Axillary
2.5	—	0.5	0.70 ± 0.19^ab^	1.80 ± 0.37^a^	1.82 ± 0.18^A^	2.04 ± 0.15^A^	0.00^a^	79.70^a^
2.5	—	1.0	1.40 ± 0.24^abcde^	2.40 ± 0.40^abc^	2.11 ± 0.11^B^	2.32 ± 0.04^AB^	0.00^a^	83.10^a^
3.0	—	0.0	2.20 ± 0.37^defg^	3.40 ± 0.24^abcd^	2.08 ± 0.18^B^	3.35 ± 0.02^DE^	0.00^a^	83.36^a^
3.0	—	0.5	2.00 ± 0.44^defg^	3.40 ± 0.24^abcd^	2.11 ± 0.06^B^	3.21 ± 0.04^DE^	0.00^a^	81.00^a^
3.0	—	1.0	1.80 ± 0.58^cdefg^	3.60 ± 0.24^bcd^	2.19 ± 0.07^BC^	3.51 ± 0.04^E^	6.80^c^	92.54^bc^
3.5	—	0.0	2.40 ± 0.58^efg^	3.60 ± 0.51^bcd^	2.21 ± 0.03^BC^	3.60 ± 0.15^E^	9.70^d^	99.60^e^
3.5	—	0.5	2.60 ± 0.24^ef^	4.00 ± 0.45^cd^	2.32 ± 0.04^BC^	4.46 ± 0.06^F^	4.24^b^	100.00^e^
3.5	—	1.0	2.80 ± 0.20^f^	5.60 ± 0.51^e^	2.40 ± 0.04^C^	5.44 ± 0.06^G^	4.10^b^	97.90^de^
4.0	—	1.0	1.80 ± 0.20^cdefg^	4.40 ± 0.24^de^	2.18 ± 0.05^BC^	4.68 ± 0.10^F^	0.00^a^	94.00^bcd^
5.0	—	1.0	0.80 ± 0.37^abc^	3.40 ± 0.51^abcd^	2.30 ± 0.04^BC^	3.25 ± 0.09^DE^	0.00^a^	83.00^a^
6.0	—	1.0	0.40 ± 0.24^a^	2.00 ± 0.84^ab^	2.25 ± 0.04^BC^	2.61 ± 0.14^BC^	0.00^a^	81.10^a^
—	1.5	1.0	1.20 ± 0.37^abcd^	2.20 ± 0.86^ab^	2.24 ± 0.04^BC^	3.20 ± 0.31^DE^	0.00^a^	83.20^a^
—	2.5	1.0	1.40 ± 0.24^abcde^	2.60 ± 0.51^abc^	2.25 ± 0.02^BC^	3.50 ± 0.42^E^	0.00^a^	81.80^a^
—	3.5	1.0	1.60 ± 0.24^bcdef^	2.80 ± 0.58^abcd^	2.31 ± 0.03^BC^	2.90 ± 0.29^DE^	0.00^a^	96.10^cde^
—	4.5	1.0	1.60 ± 0.24^bcdef^	3.40 ± 0.51^abcd^	2.26 ± 0.04^BC^	3.68 ± 0.14^E^	0.00^a^	91.10^b^

Mean ± SE, *n *= 10. Different letters indicate significant differences between media at *P *= 0.05.

**Table 4:** Effects of different concentrations and combinations of cytokinin and auxin on shoot production from two different sources of explants in red *C. Crotalifera *(CCR).

BAP (mg/L)	Kinetin (mg/L)	NAA (mg/L)	Number of shoot**s**		Height of shoot (cm)		Percentage of leaf (%)	
			Apical	Axillary	Apical	Axillary	Apical	Axillary
2.5	—	0.5	1.00 ± 0.00^abc^	1.80 ± 0.20^a^	1.80 ± 0.20^A^	2.13 ± 0.03^A^	0.00^a^	81.10^a^
2.5	—	1.0	1.00 ± 0.00^abc^	2.20 ± 0.20^a^	1.90 ± 0.11^AB^	2.25 ± 0.02^AB^	0.00^a^	83.10^ab^
3.0	—	0.0	0.90 ± 0.24^abc^	2.00 ± 0.00^a^	2.22 ± 0.09^CDEF^	3.64 ± 0.10^D^	4.30^b^	83.36^ab^
3.0	—	0.5	1.20 ± 0.37^abcd^	2.40 ± 0.40^a^	2.11 ± 0.03^BCDE^	3.63 ± 0.10^D^	0.00^a^	83.66^ab^
3.0	—	1.0	1.20 ± 0.37^abcd^	2.20 ± 0.37^a^	2.31 ± 0.08^DEF^	4.47 ± 0.16^E^	6.80^c^	91.54^cd^
3.5	—	0.0	1.60 ± 0.24^cd^	3.60 ± 0.40^bc^	2.28 ± 0.07^DEF^	4.49 ± 0.23^E^	9.70^d^	99.60^f^
3.5	—	0.5	1.60 ± 0.24^cd^	3.80 ± 0.37^bc^	2.35 ± 0.05^EF^	4.63 ± 0.17^EF^	4.74^b^	100.00^f^
3.5	—	1.0	2.00 ± 0.32^d^	4.40 ± 0.24^c^	2.40 ± 0.04^F^	4.85 ± 0.12^F^	4.30^b^	98.80^ef^
4.0	—	1.0	1.40 ± 0.24^bcd^	3.40 ± 0.51^b^	2.04 ± 0.05^ABCD^	3.06 ± 0.16^C^	0.00^a^	93.70^de^
5.0	—	1.0	0.60 ± 0.24^ab^	2.00 ± 0.32^a^	1.99 ± 0.06^ABC^	3.24 ± 0.07^C^	0.00^a^	83.00^ab^
6.0	—	1.0	0.40 ± 0.24^a^	1.60 ± 0.24^a^	1.96 ± 0.12^ABC^	2.31 ± 0.06^AB^	0.00^a^	81.10^a^
—	1.5	1.0	1.20 ± 0.20^abcd^	2.00 ± 0.00^a^	2.07 ± 0.04^ABCD^	3.16 ± 0.05^C^	0.00^a^	83.26^ab^
—	2.5	1.0	1.40 ± 0.24^bcd^	2.00 ± 0.32^a^	2.04 ± 0.04^ABCD^	3.22 ± 0.03^C^	0.00^a^	81.86^a^
—	3.5	1.0	0.60 ± 0.24^ab^	1.80 ± 0.37^a^	2.16 ± 0.05^BCDEF^	2.56 ± 0.12^B^	0.00^a^	85.00^ab^
—	4.5	1.0	1.40 ± 0.24^bcd^	1.80 ± 0.20^a^	2.09 ± 0.06^BCDE^	3.19 ± 0.12^C^	0.00^a^	88.10^bc^

Mean ± SE, *n *= 10. Different letters indicate significant differences between media at *P *= 0.05.

**Table 5:** Effects of apex damage on production of microshoots in *C. crotalifera* (CCR and CCY).

Apex damage	Shoot production (%)		Number of microshoots		Height of shoot (cm)	
	CCY	CCR	CCY	CCR	CCY	CCR
Longitudinal section	100.0	100.0	8.0 ± 0.9	8.7 ± 0.8	4.0 ± 0.5	3.9 ± 0.3
Cross section	50.0	50.0	1.0 ± 0.3	0.9 ± 0.3	0.7 ± 0.1	0.5 ± 0.1

**Table 6:** Modification of multiplication media with different concentrations of activated charcoal and glutamine.

Media	Treatments
3510	MS + 3.5 mg/L BAP + 1.0 mg/L NAA (control)
MSC1	MS + 1.0 g/L activated charcoal
MSC2	MS + 2.0 g/L activated charcoal
MSC3	MS + 3.0 g/L activated charcoal
3510 G03	MS + 3.5 mg/L BAP + 1.0 mg/L NAA + 0.3 g/L glutamine
3510 G05	MS + 3.5 mg/L BAP + 1.0 mg/L NAA + 0.5 g/L glutamine
3510 G10	MS + 3.5 mg/L BAP + 1.0 mg/L NAA + 1.0 g/L glutamine
3510 G20	MS + 3.5 mg/L BAP + 1.0 mg/L NAA + 2.0 g/L glutamine
3510 1C	MS + 3.5 mg/L BAP + 1.0 mg/L NAA + 1.0 g/L activated charcoal
3510 G1C	MS + 3.5 mg/L BAP + 1.0 mg/L NAA + 1.0 g/L activated charcoal + 1.0 g/L glutamine

**Table 7:** Effects of activated charcoal and glutamine on concentration of leaf pigments in yellow *C. crotalifera* (CCY).

Media	Chlorophyll a (*µ*g/mL)	Chlorophyll b (*µ*g/mL)	Carotenoid (*µ*g/mL)
3510	10.873 ± 0.256^a^	11.194 ± 0.069^A^	4.695 ± 0.073^a^
MSC1	20.386 ± 1.349^d^	13.801 ± 0.508^D^	7.050 ± 0.596^c^
MSC2	17.030 ± 0.702^c^	12.713 ± 0.165^C^	6.274 ± 0.225^bc^
MSC3	16.830 ± 0.553^c^	12.513 ± 0.243^BC^	6.074 ± 0.175^b^
3510 G03	11.430 ± 0.591^a^	11.755 ± 0.331^ABC^	4.914 ± 0.167^a^
3510 G05	11.687 ± 0.728^a^	11.389 ± 0.160^AB^	4.805 ± 0.210^a^
3510 G10	14.056 ± 0.131^b^	11.882 ± 0.085^ABC^	5.535 ± 0.052^ab^
3510 G20	14.016 ± 0.120^b^	11.662 ± 0.175^ABC^	5.315 ± 0.173^ab^
3510 1C	21.520 ± 1.116^d^	15.025 ± 0.766^E^	7.990 ± 0.536^d^
3510 G1C	22.380 ± 0.839^d^	15.825 ± 0.492^E^	8.190 ± 0.433^d^

Mean ± SE, *n *= 10. Different letters indicate significant differences between media at *P *= 0.05.

**Table 8:** Effects of auxin on roots induction in yellow *C. crotalifera* (CCY).

NAA (mg/L)	IBA (mg/L)	Number of root**s**	Length of root (cm)	Diameter of root (cm)
0.00	0.00	2.60 ± 0.40^ab^	4.26 ± 0.31^AB^	0.15 ± 0.01^cd^
0.10	—	2.80 ± 0.37^ab^	5.94 ± 0.63^BC^	0.15 ± 0.01^cd^
0.50	—	3.60 ± 0.24^bc^	7.02 ± 0.28^CD^	0.18 ± 0.01^de^
1.00	—	6.40 ± 0.24^d^	2.84 ± 0.12^A^	0.20 ± 0.01^e^
1.50	—	4.00 ± 0.32^c^	2.91 ± 0.39^A^	0.20 ± 0.01^e^
—	0.10	2.00 ± 0.32^a^	7.00 ± 1.17^CD^	0.09 ± 0.01^a^
—	0.50	1.80 ± 0.20^a^	8.24 ± 0.26^D^	0.11 ± 0.04^ab^
—	1.00	3.20 ± 0.37^bc^	5.32 ± 0.86^BC^	0.13 ± 0.01^bc^
—	1.50	2.80 ± 0.37^ab^	5.20 ± 0.73^BC^	0.13 ± 0.01^bc^

Mean ± SE, *n *= 10. Different letters indicate significant differences between media at *P *= 0.05.

**Table 9:** Stomata morphology of *in vitro* and acclimatized (*in vivo*) plantlets of yellow *C. crotalifera* (CCY) under scanning electron microscope.

Physiology	Adaxial surface		Abaxial surface	
	*In vitro *	*In vivo *	*In vitro *	*In vivo *
Mean number stomata (per 100 mm^2^)	5.75 ± 1.25	6.50 ± 0.50	20.25 ± 2.25	22.00 ± 2.11
Length (*µ*m)	27.65 ± 1.00	26.18 ± 0.72	25.95 ± 0.70	24.28 ± 0.82
Width (*µ*m)	11.15 ± 0.26	9.73 ± 0.18	9.39 ± 0.49	8.71 ± 0.13
